# A Bayesian Approach to Estimate the Prevalence of *Schistosomiasis japonica* Infection in the Hubei Province Lake Regions, China

**DOI:** 10.3390/ijerph10072799

**Published:** 2013-07-05

**Authors:** Xin Xia, Hui-Ping Zhu, Chuan-Hua Yu, Xing-Jian Xu, Ren-Dong Li, Juan Qiu

**Affiliations:** 1 School of Public Health & Global Health Institute, Wuhan University, No. 115, Donghu Road, Wuhan 430071, China; E-Mail: xiaxin84@163.com; 2 School of Public Health, Beijing Municipal Key Laboratory of Clinical Epidemiology, Capital Medical University, No. 10, Xitoutiao, Youanmen, Beijing 100069, China; E-Mail: zhuhuiping@ccmu.edu.cn; 3 Institute of Schistosomiasis Control, Hubei Provincial Center for Disease Control, No. 6, Zhuodaoquan Road, Wuhan 430079, China; E-Mail: xuxj0612@yahoo.com.cn; 4 Institute of Geodesy and Geophysics, Chinese Academy of Science, No. 136, Donghu Road, Wuhan 430077, China; E-Mails: lrd@asch.whigg.ac.cn (R.-D.L.); qiujuan2011@gmail.com (J.Q.)

**Keywords:** *Schistosomiasis japonica*, Bayesian estimation, prevalence, IHA, Kato-Katz

## Abstract

A Bayesian inference model was introduced to estimate community prevalence of *Schistosomiasis japonica* infection based on the data of a large-scale survey of *Schistosomiasis japonica* in the lake region in Hubei Province. A multistage clusterrandom sampling approach was applied to the endemic villages in the lake regions of Hubei Province in 2011. IHA test and Kato-Katz test were applied for the detection of the *S. japonica* infection in the sampled population. Expert knowledge on sensitivities and specificities of IHA test and Kato-Katz test were collected based on a two-round interview. Prevalence of *S. japonica* infection was estimated by a Bayesian hierarchical model in two different situations. In Situation 1, Bayesian estimation used both IHA test data and Kato-Katz test data to estimate the prevalence of *S. japonica*. In Situation 2, only IHA test data was used for Bayesian estimation. Finally 14 cities and 46 villages from the lake regions of Hubei Province including 50,980 residents were sampled. Sensitivity and specificity for IHA test ranged from 80% to 90% and 70% to 80%, respectively. For the Kato-Katz test, sensitivity and specificity were from 20% to 70% and 90% to 100%, respectively. Similar estimated prevalence was obtained in the two situations. Estimated prevalence among sampled villages was almost below 13% in both situations and varied from 0.95% to 12.26% when only using data from the IHA test. The study indicated that it is feasible to apply IHA test only combining with Bayesian method to estimate the prevalence of *S. japonica* infection in large-scale surveys.

## 1. Introduction

*Schistosomiasis japonica* is a major public health problem in China. In the past 60 years, China has made great efforts to control this serious endemic disease and made remarkable achievements. The number of infected people has decreased significantly, from 11.6 million in the 1950s to approximately 0.73 million in 2004 [1,2,3]. Data from the latest national epidemiologic survey showed that the average prevalence rate was 2.5% in all selected endemic areas and 5.1% in the areas where the transmission of *S. japonica* is uncontrolled [4]. In order to thoroughly eliminate *S. japonica*, a national program has conducted since 2004, which aims to control the prevalence of *S. japonica* infection below 5% in 2008 and 1% in 2015 in all epidemic communities [5]. To achieve this goal, estimating the real prevalence of *S. japonica* by simple and accurate diagnostic tests is of supreme importance, especially in areas of low prevalence.

In recent years, a large number of studies have focused on studying the diagnostic accuracy of *S. japonica*, prevalence estimates, which was mostly based on the assumption that the Kato-Katz result was the “gold standard” for the diagnosis of *S. japonica* infection [6,7,8,9]. However, with the decreasing intensity of transmission in endemic areas, this “gold standard” was not as precise and appropriate enough in low prevalence regions as it was in moderate and severe endemic areas [10,11,12]. Also, the sensitivity and specificity of immunodiagnostic tests like indirect hemagglutination assay (IHA) were decreasing in low prevalence of *S. japonica* infection areas, because most false negative results were obtained from patients with very low infection intensities (generally less than 20 eggs per gram of feces) by diagnosing the serum specimens [8,13]. Since sensitivity of test methods can be easily affected by certain factors like intensity of transmission, region and reagent type, serum reagents need to be standardized and test properties are supposed to be estimated taking into account these potential impact factors. Although IHA is widely used, it cannot distinguish current and cured infections, so additional Kato-Katz testing is typically relied upon.

Taking into account the fact that both Kato-Katz and IHA tests are already being used extensively, more appropriate statistical methods are urgently needed to deal with the disadvantages occurring in both tests. Bayesian analysis has been widely used in the research of parasitic disease, especially for prevalence estimation, as it can integrate both sensitivity and specificity of tests into analysis to improve the accuracy of estimation when prior information is available [7,10,11,12]. The two tests combined with Baysesian estimation can produce an estimate of *S. japonica* prevalence. The present study aims to estimate the prevalence of *S. japonica* infection in the lake region of Hubei Province by applying Bayesian methods in two situations. In Situation 1, Bayesian estimation used both IHA test data and Kato-Katz test data to estimate the prevalence of *S. japonica*. In Situation 2, only IHA test data was used for Bayesian estimation. We compared the prevalence of *S. japonica* infection in these two situations to verify whether it’s feasible for us to only use the IHA test to estimate the prevalence infection in *S. japonica* endemic areas.

## 2. Methods

### 2.1. Ethics Statement

Informed written consent was obtained from participants or a legal guardian of children aged under 18-years old. This study was approved by the Ethics Committee of School of Public Health, Wuhan University.

### 2.2. Kato-Katz and IHA

The Kato-Katz technique is a commonly used test for detecting schistosome eggs [14], because it is comparatively inexpensive, quantitative and easy to use. It is considered to be the reference standard for diagnosis of *S. japonicum* infections [13]. However, the sensitivity of Kato-Katz could be decreased in areas of low endemicity, especially for individuals with low egg burdens. Repeated stool collection and examination can improve the sensitivity of the Kato-Katz assay, but this process is more costly and labor intensive [15].

The IHA test is a kind of immunological techniques for *S. japonicum* antibody detection [16]. It is the most widely used assay in China because of its lack of technical problems, higher sensitivity and ease of use over stool examination. The IHA test is based on an indicator system that is composed of sheep red blood cell or human red blood cell with “O” blood type coated with soluble antigen extracted from *S. japonicum* eggs. More details about the IHA test can be found in previous studies [10,16].

### 2.3. Investigation of S. japonica Prevalence

**Figure 1:**
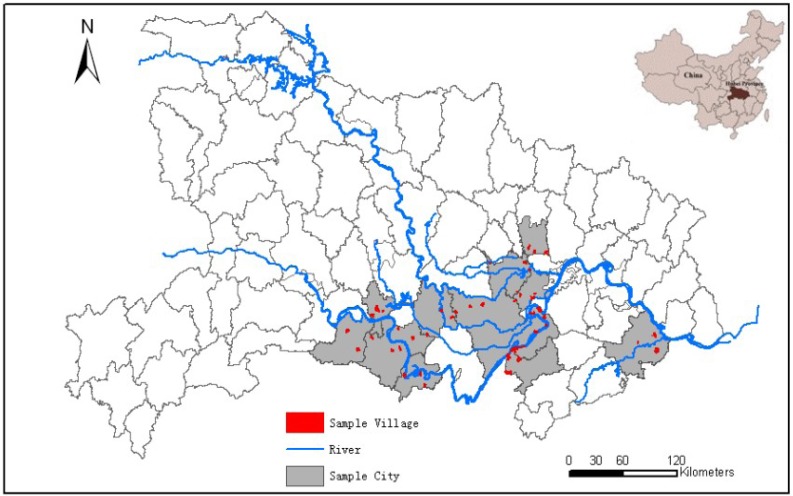
Sampled distribution of the study cities and villages in the lake region, Hubei Province, China.

A multistage cluster random sampling approach was applied to the endemic villages in the lake regions of Hubei Province in 2011 (Figure 1). In the first stage, 14 cities were randomly sampled from the formerly endemic lake regions. In the second stage, a cluster random sampling approach was applied and at least three villages were selected from each city. A random number table was applied at the beginning of the study to ensure cities and villages were randomly selected into the study after each sampled unit was assigned a specific number. In principle, it was required that the population of a sampled village should not be less than 1,000 residents, and all residents aged 5 to 65 years in a sampled village were asked to participate in the survey to ensure that the prevalence estimation was valid. In the present study, IHA examination was firstly used for the serological screening test for all sampled populations and the Kato-Katz test was then applied for individuals with positive IHA test results. The test results (positive or negative) of IHA and Kato-Katz were analyzed in our study. According to the definition of combined diagnostic for *schistosomiasis* infection [6,8,10], the prevalence of *S. japonica* was the proportion of people who are positive on both tests.

### 2.4. Sensitivities and Specificities of the Tests

Expert knowledge on the sensitivities and specificities of the IHA and the Kato-Katz test were collected through a two-round interview based on questionnaires. Experts from Institute of Parasitic Diseases, Centers of Disease Control and medical universities provided their estimated values including the average, minimum, maximum value of the sensitivity and specificity of IHA and the Kato-Katz test according to the questionnaire in the first round. Medians of the minima and maxima were computed for both the sensitivity and specificity of each test to form the range of the sensitivity and specificity for each test, and this range were sent back to experts using a semi-structured questionnaire for reference. In the second round, experts were asked to make a choice whether they agree with these given values. If not, they would be further asked to provide a reasonable 95% CI values in their opinions. Finally, we calculated the 95% Confidence Interval of the medians of values as prior ranges of test properties for Bayesian analysis.

### 2.5. Statistical Analysis

#### 2.5.1. Traditional Statistics

Descriptive statistics were summarized for the population in each selected city and the prevalence of *S. japonica* infection using the combined diagnostic was calculated.

#### 2.5.2. Prior Distribution

We assumed the prior distributions of the sensitivity and specificity of each test from expert knowledge were beta (α, β) distributions in the present study [17]. We set *θ* to be the prior mean value, which matched with the center of the range of the each test property, and set *S_θ_* to be the prior standard deviation, which matched with one quarter of the range of each test property. Then we can calculate α and β values using the following formulae [18,19]:

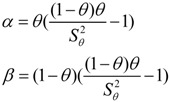



In addition, we assumed that we didn’t have any prior information of prevalence, which means we assumed the prior distribution of prevalence follows the beta(1,1) distribution.

#### 2.5.3. Bayesian Statistics

A Bayesian Hierarchical model was applied to estimate the prevalence of *S. japonica* infection for each sampled village and resident city in two different situations, including using data from the combined IHA and Kato-Katz data, and data from the IHA test alone.

In Situation 1, data from the IHA and Kato-Katz combined diagnostic test was used to estimate the prevalence of *S. japonica* in each village as well as in each city on the assumption that the sensitivity and specificity of IHA and Kato-Katz test were the same in all villages. Independence between IHA and Kato-Katz test was also assumed due to the different detection techniques [8]. For the *k*th village:

**Table 1:** Cross-classified data of IHA test and Kato-Katz test for the *k*th village.

IHA	Kato-Katz		Total
	+	−	
+	*t_2k_*	*t_1k_* − *t_2k_*	*t_1k_*
–			*n_1k_* − *t_1k_*
Total			*n_1k_*

Data are consistent with the following distributional assumptions:

t*_1k_* ~ Binomial(p*_1k_*, n*_1k_*)

t*_2k_* ~ Binomial(p*_2k_*, n*_2k_*)

Among the above:

p*_1k_* = *Se_1_* × *π_k_* + (1 − *Sp_1_*) × (1 − *π_k_*)

p*_2k_* = *Se_1_* × *Se_2_* × *π_k_* + (1 − *Sp_1_*) × (1 – *Sp_2_*) × (1 − *π_k_*)/p*_1k_*

where n*_1k_*, t*_1k_*, n*_2k_* and t*_2k_* represent the number examined using IHA, number of positive IHA tests, the number of Kato-Katz examinations and number of positive Kato-Katz tests of the *k*th village (see Table 1), p*_1k_*, p*_2k_* and *π_k_* represented seroprevalence, prevalence of Kato-Katz in the positive IHA population and the prevalence of population. *Se_1_* and *Sp_1_* represented the sensitivities and specificities of IHA, *Se_2_* and *Sp_2_* represented the sensitivities and specificities of the Kato-Katz test. In theory, every positive IHA case would be examined by the Kato-Katz test, which means n*_2k_* should equal to t*_1k_*, while in fact, some positive IHA cases were not followed up and examined by the Kato-Katz method, therefore n*_2k_* was less slightly than t_1*k*_ in our study.

Random effects including city and village were included in the model to reflect the data structure of the sampling method in this study:

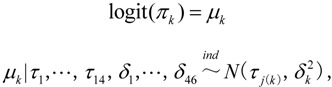

where 1 ≤ *j(k)* ≤ 14 is the city *j* associated with village *k*:

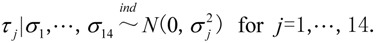

where *μ_k_* and *τ_j_* represented the random effects of the village and city respectively and they were interpreted as the variation of prevalence of each village and each city. 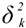 and 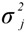 accordingly represented the variance. The prevalence of *S. japonica* infection in each city could be calculated according to the random effects *τ_j_* as follows:

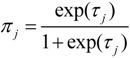



In Situation 2, the model assumption and the random effect were the same as those in Situation 1 except only data concerning the IHA test were used in this situation. In addition, formulae related to t*_2k_* and p*_2k_* are not needed. *We assumed a non-informative prior distribution and inverted gamma distribution for the variance (i.e., 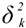 and 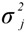) of each random effect.*

Models were fitted in WinBUGS 1.4.1 and Markov chain Monte Carlo (MCMC) was employed to estimate all parameters [9]. Model convergence was evaluated by examining time series plot for each parameter and the Gelman-Rubin statistic. Finally, a sensitivity analysis was conducted by changing the prior parameters to check whether similar results were seen.

## 3. Results

**Figure 2:**
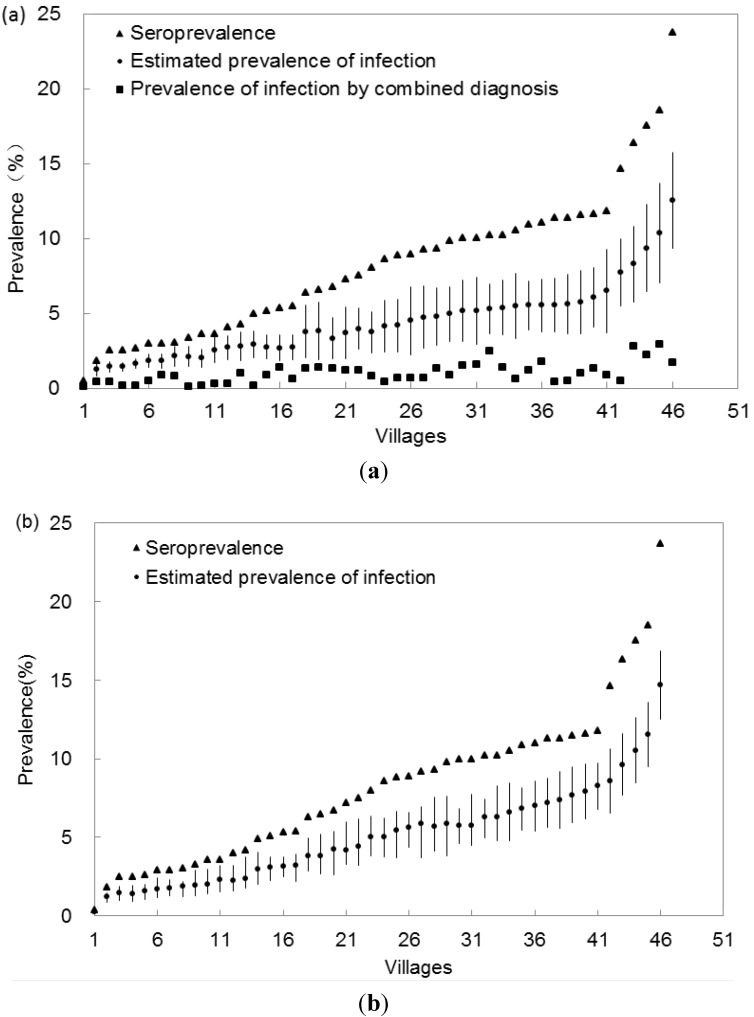
Estimated prevalence of infection for each village in the survey of *S. japonica* in Hubei Province, China, 2011. (**a**) Prevalence of infection was estimated using data from IHA test and Kato-Katz test. (Situation 1). (**b**) Prevalence of infection was estimated using data from the IHA test alone (Situation 2). Villages were ordered by ascending seroprevalence.

In total, 46 villages in 14 cities of China with 50,980 residents located in the lake regions were sampled. In the end, 93.1% of the residents (47,463/50,980) agreed to participate in the survey and received the IHA test examination. The total number of positive IHA tests was 3,912, of which, 89.8% (3,532/3,912) of the residents were then examined by the Kato-Katz test. The seroprevalence (*i.e*., via IHA testing) of *S. japonica* infection ranged from 1.3% to 15.8% for all sampled cities, 0.4% to 23.7% for all sampled villages. The prevalence of *S. japonica* infection by combined diagnostic tests was 0.3% to 2.1% for all sampled cities, and 0.1% to 2.9% for all sampled villages. The average seroprevalence was 8.2% and the average prevalence by combined diagnostic was 1.0% (see Table 2 and Figure 2(a)).

**Table 2:** Prevalence of 14 cities by diagnosis of IHA and Kato-Katz in the survey of *S. japonica* in Hubei Province, China, 2011.

City	IHA Test			Kato-Katz Test		
	Detected Numbers	Positive Number	seroprevalence^ a^	Detected Numbers	Positive Number	Prevalence of infection ^b^
Caidian [1]	964	13	1.3	13	3	0.3
Chibi [2]	1,002	145	8.7	138	12	2.1
Gongan [3]	5,657	56	15.8	42	4	1.1
Hanchuan [4]	5,193	413	8.0	385	51	1.0
Honghu [5]	6,408	124	9.3	80	9	1.2
Jiayu [6]	1,592	894	5.2	880	63	1.1
Jiangling [7]	2,449	248	10.1	235	32	1.4
Jingzhou [8]	1,562	395	8.0	362	40	0.9
Qianjiang [9]	5,019	596	8.1	555	71	0.8
Shishou [10]	3,115	266	12.7	175	31	1.4
Songzi [11]	3,041	83	8.7	81	17	1.6
Xiantao [12]	6,823	87	2.7	38	9	0.8
Xiaonan [13]	915	182	6.1	177	51	0.5
Yangxin [14]	3,723	408	3.9	371	38	0.3
Total	47,463	3,912	8.2	3,532	432	1.0

^a^ seroprevalence = (positive number of IHA /detected number of IHA);^ b^ prevalence = (positive number of IHA /detected number of IHA) × (positive number of Kato-Katz/ detected number of Kato-Katz test).

Eighteen experts were invited and 15 experts participated in this survey in both rounds. Table 3 shows the prior distributions of the sensitivity and specificity of the IHA test and the Kato-Katz test according to expert knowledge. The final 95% CI of sensitivity and specificity were (0.80, 0.90) and (0.70, 0.80) for the IHA, (0.20, 0.70) and (0.90, 1.00) for the Kato-Katz test, respectively. Parameters of the beta(α, β) prior distributions calculated upon the sensitivity and specificity of each test are shown in Table 2.

**Table 3:** Parameters of the beta(α, β) prior distributions for the sensitivities and specificities of IHA and Kato-Katz tests for *S. japonica* examination in the lake regions in Hubei, China.

Test	Sensitivity			Specificity		
	Range	α	β	Range	α	β
IHA	0.80–0.90	172.55	30.45	0.70–0.80	224.25	74.75
Kato-Katz	0.20–0.70	6.68	8.16	0.90–1.00	71.25	3.75

Figure 2 presents the median with 95% confidence intervals of estimated prevalence of *S. japonica* infection for each village in Situation 1 and Situation 2, which are generalized in Table 4. The posterior medians of the prevalence in the 46 sampled villages ranged from 0.32% to 12.13% with a median of 3.72% in Situation 1 and from 0.95% to 12.26% with a median of 4.50% in Situation 2. All estimated prevalence was smaller than the seroprevalence in the two situations (upper confidence interval was smaller than the corresponding seroprevalence). The estimated prevalence was higher than that calculated by the combined diagnostics in Situation 1. After compaing the 95% confidence intervals of estimated prevalence in the two situations (data not showed), found that the estimated prevalence in both situations appear be not significantly difference for almost all sampled villages (95% CI in the two situations overlapped, see Figure 2).

**Table 4:** Bayesian prevalence estimates (posterior median) of *S. japonica* infection within villages and cities, respectively, in the survey of *S. japonica* in Hubei Province, China, 2011.

	Min	Q1	Median	Q3	Max
***Village prevalence (%)***					
Situation 1 ^a^	0.32	2.39	3.72	5.23	12.1
Situation 2 ^b^	0.95	2.07	4.5	5.79	12.3
***City prevalence (%)***					
Situation 1 ^a^	2.39	2.51	3.54	4.17	6.72
Situation 2 ^b^	1.52	2.41	4.06	4.72	7.26

^a^ Prevalence of infection was estimated using data from the IHA test and Kato-Katz test. ^b^ Prevalence of infection was estimated using data from the IHA test alone.

**Figure 3:**
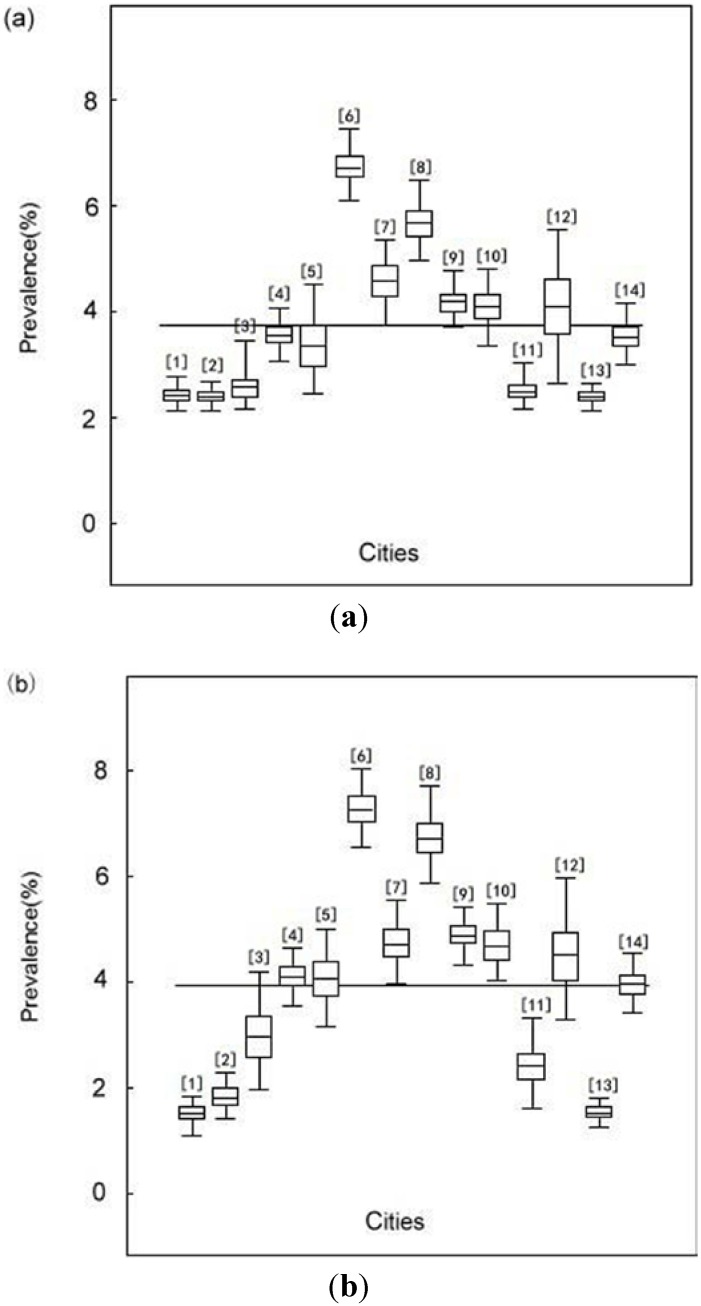
Estimated prevalence of infection in each sampled city in the survey of *S. japonica* in Hubei Province, China, 2011. (**a**) Prevalence of infection was estimated using data from the IHA test and Kato-Katz test (Situation 1). (**b**) Prevalence of infection was estimated using data from the IHA test alone (Situation 2).

Figure 3 presents the median with 95% confidence intervals of estimated prevalence of *S. japonica* infection for sampled 14 cities in Hubei province in Situation 1 and Situation 2, which were also summarized in Table 3. The posterior medians of the prevalence in 14 sampled cities ranged from 2.39% to 6.71% with a median of 3.54% in Situation 1 and from 1.52% to 7.26% with a median of 4.06% in Situation 2. The estimated prevalence for each city showed no significant difference between two situations since the 95% confidence intervals overlapped each other except that when estimated prevalence was extremely low (less than 2% as showed in the Figure 3). Sensitivity analysis results showed that the 95% confidence intervals of the prevalence were much broader when the range of the test properties were enlarged (table not shown).

## 4. Discussion and Study Limitations

The survey of *S. japonica* infection in the present study was conducted in low endemic areas and most previous studies were conducted in moderate and severe prevalence areas [6,12,20], therefore, the value of sensitivity and specificity of each test in previous publications were not adequate for the present study. Expert knowledge was then applied to provide accuracy to the test properties in this study. Experts would consider the potential impacts of factors on the accuracy of tests properties in the course of evaluating the value of sensitivity and specificity to ensure a relatively accurate range was obtained. Finally, the range of values of sensitivity and specificity we obtained by expert knowledge were narrower than those reported in the previous researches [6].

It’s difficult to obtain accurate prevalence levels by simply computing the ratio of the total number of positive cases among all selected subjects due to decreasing detection accuracy of standard diagnostic methods in low endemic areas. The Bayesian method has been successfully applied to estimate population prevalence and test properties in *S. japonica* researches when a “gold standard” is absent [4,19,21]. Traditionally, test properties were treated as constants for prevalence estimation, which led to the variability of prevalence estimates to be unavoidably underestimated [19]. The Bayesian approach treated the uncertainty of sensitivity and specificity as variable parameters and incorporated prior information into the prevalence estimation. In our study, we treated sensitivity and specificity of each test as variable parameters and brought them into the Bayesian models to estimate the prevalence of *schistosomiasis* infection. Compared with traditional methods, we could obtain a more accurate estimated prevalence by introducing prior information into the Bayesian model [18,21,22]. Sensitivity analysis results of this study supported the finding of previous research that when the range of test properties widened, 95% confidence intervals for the estimate prevalence of *S. japonica* infection becomes much broader [23]. We also used non-informative prior distributions for sensitivity and specificity obtaining broader 95% confidence intervals. Sensitivity analysis indicates that the estimated prevalence value were dependent to a certain extent on the prior information of test properties.

Previous research found that if the number of parameters estimated in a model is larger than the degrees of freedom measured by the data, then model non-identifiability occurs. As Branscum *et al.* and Toft *et al.* found [24,25], however, the problem mentioned above can be alleviated on the assumption that the test properties are the same among several different populations when having more than one population in a model, then the degrees of freedom will at least equal to the number of estimated parameters. In the present study, we hypothesized that the test properties were the same among all selected villages within each city in order to cut down the number of parameters and the prior distributions of test properties were derived from the experienced experts’ knowledge. Therefore, we elicited reliable prevalence estimates.

In the present study, we obtained similar prevalence estimates of *S. japonica* infection in the two situations, which means that it’s feasible to employ only IHA instead of a combination of IHA and the Kato-Katz test to estimate the prevalence of *S. japonica* infection. Compared with the Kato-Katz test, IHA could improve the compliance of residents, save costs and reduce the manpower needs in a large-scale survey.

Like all research, this study has some limitations. First, our analysis was based on the assumption that the sensitivity and specificity of each diagnostic test were the same in all villages. However, the accuracy of the diagnostic test may vary in different regions because of the impacts of intensity of transmission, region and reagent’s type. In our study, the sampled villages were close in terms of geographical environment and degree of infection for *S. japonica* and the reagents used for detection were also the same. All these factors mentioned above can reduce the difference of the accuracy of the detection method. Second, the estimated accuracy of prevalence of *S. japonica* infection mostly relied on the accuracy of the sensitivity and specificity of each test, but we couldn’t obtain an absolutely accurate range of the sensitivity and specificity of each diagnostic test in present study since the test properties would be easily affected by external factors. We chose to evaluate the test properties of the IHA and Kato-Katz tests by expert knowledge to obtain a reasonably accurate range value to estimate the prevalence of *S. japonica* infection and the results in the present study appear good. Third, in a large-scale survey, missing data is an inevitable phenomenon that may have an impact on the precision of the prevalence estimation of disease. In our study, nearly 9.7% residents taking the IHA test didn’t receive the Kato-Katz test, which was largely because some residents refused to provide stools, and some residents were absent during the period of stool sample collection. The missing data might affect our results to a certain extent, but the bias is likely slight because the proportion in such a large-scale survey was not big, and Bayesian statistics can deal with this kind of missing data. Fourth, when building the Bayesian model, we made the independence assumption of the tests due to the different detection techniques in our study, while Hanson *et al*. [26] found extreme dependence among serological and stool-based tests. The assumption of conditional dependence can severely bias prevalence estimates when independent assumption is not true. However, it’s difficult to determine the conditional dependence relationship between IHA and Kato-Katz in the absence of a diagnostic gold standard. The positive IHA test cases showed an extremely low probability of infection of *Schistosomiasis japonica*, and the specificity of the IHA test was high. We could basically ignore the Kato-Katz test effect when we got negative results by the IHA test in our study and made the independence assumption. However, to some extent, the independence assumption was somewhat subjective and performing a model selection based on the data to determine the model could improve future analysis.

## 5. Conclusions

Despite these limitations, we can still draw the conclusion that the Bayesian approach can be applied to estimate the prevalence of *S. japonica* infection when parasitological techniques serving as “gold standard” are not precise in low endemic areas. It’s possible for us to only apply the IHA test combined with the Bayesian method for the estimation of the prevalence of *S. japonica* infection in large-scale surveys in future.
